# Qualitative Study of Changes in Alcohol Use Among HIV-Infected Adults Entering Care and Treatment for HIV/AIDS in Rural Southwest Uganda

**DOI:** 10.1007/s10461-014-0918-5

**Published:** 2014-10-17

**Authors:** Radhika Sundararajan, Monique A. Wyatt, Sarah Woolf-King, Emily E. Pisarski, Nneka Emenyonu, Winnie R. Muyindike, Judith A. Hahn, Norma C. Ware

**Affiliations:** 1 Department of Emergency Medicine, University of California, San Diego, CA USA; 2 Department of Global Health and Social Medicine, Harvard Medical School, Boston, MA USA; 3 Department of Medicine, University of California, San Francisco, CA USA; 4 Immune Suppression Syndrome Clinic, Mbarara University of Science and Technology, Mbarara, Uganda

**Keywords:** HIV/AIDS, Uganda, Alcohol use, Behavior change

## Abstract

Alcohol has a substantial negative impact on the HIV epidemic in sub-Saharan Africa, particularly in Uganda, where heavy alcohol consumption is common. Using a content analytic approach, this qualitative study characterizes changes in alcohol use among 59 HIV-infected Ugandan adults (>18 years old), who reported any alcohol use in the previous year as they entered HIV care. Most participants reported attempting to cease or reduce alcohol intake over the study period. Reasons for decreased use included advice from clinicians, interference with social obligations, threats to financial security, and negative impact on social standing. Participants reported difficulty abstaining from alcohol, with incentives to continue drinking including desire for social inclusion, stress relief, and enjoyment of alcohol. These contrasting incentives created a moral quandary for some participants, who felt ‘pulled’ between ‘good’ and ‘bad’ influences. Results suggest brief interventions addressing self-identified obstacles to change may facilitate long-term reductions in drinking in this population.

## Introduction

Alcohol use has been described as “adding fuel to the fire” of the HIV epidemic in sub-Saharan Africa [1]. Alcohol-associated high-risk sexual behavior [2–7], decreased adherence to antiretroviral therapy (ART) [8–11], and possibly, a direct biological impact of alcohol use on progression of HIV disease [12–19] have all been cited as contributing factors.

In Uganda, the current prevalence of HIV infection is 7.2 %, a reversal of the declines in prevalence seen from 1992 to 2005 [20]. ART was introduced in Uganda during the mid-1990s. Uganda was among four countries to pilot the UNAIDS HIV Drug Access Initiative in 1998, which aimed to increase access to HIV treatment by making it available at lower prices [21]. Currently, about 400,000 Ugandan adults are taking ART [20]. This will likely increase with the recent revisions to Uganda’s national ART eligibility guidelines, which raise the threshold for ART initiation to a CD4 count of 500 cells/mm^3^ [22, 23].

While more than half (56 %) of Ugandans abstain from alcohol, among those who drink, heavy alcohol consumption is very common. Among male drinkers, the per capita yearly rate of pure alcohol consumption is 25.6 l; among female drinkers, it is 19.6 l [24]. These are among the highest rates of alcohol consumption in the world. The most common beverages consumed are beer, made commercially in Uganda and imported from Europe, and a gin known as waragi, made from bananas. Ugandans also drink locally produced, traditional alcohols made from grains, sorghum, and fruit. The combination of common heavy alcohol use and high HIV prevalence in Uganda increases the risk of ongoing transmission of HIV in Uganda, underscoring the need for research to develop alcohol interventions.

Substantial decreases in self-reported alcohol consumption in the absence of systematic intervention have recently been documented for HIV-infected populations in two separate studies in Uganda [25, 26]. One of these studies found that hazardous alcohol consumption (defined as scoring ≥3 for women and ≥4 for men on the alcohol use disorders identification test-consumption, AUDIT-C) [27] declined from 35 to 16 % among baseline drinkers in the three months following HIV counseling and testing at a large urban hospital in Kampala [25]. The other found that in a cohort of persons initiating ART, among those reporting any alcohol use in the three months prior to baseline, 83 % became abstinent for at least 3 months during follow up, with a median duration of abstinence of 3.25 years [26]. Additional research is required to delineate the reasons (i.e., motives) for these reductions, in order to translate these data into sustainable alcohol interventions that complement existing HIV care in the region.

Motives to drink and to abstain have been well characterized in resource rich countries [28–31]. Several studies have revealed that alcohol is consumed in order to cope with stress, enhance positive mood, attenuate negative emotions, obtain social rewards, and avoid social rejection [31]. Research on HIV-positive adults in primary care settings in the United States has further shown that motives to limit alcohol consumption among people living with HIV are largely similar to those of the general population, centering on concerns about the effects of drinking to intoxication and the consistency of drinking with lifestyle choices (e.g., religious beliefs) [32]. This research also revealed that, unique to HIV-positive adults, social issues related to drinking are endorsed both as reasons *for* (e.g., responding to social pressure) and *against* (e.g., maintaining social responsibilities, avoiding social disapproval) alcohol use, underscoring the need to include samples of exclusively HIV-positive adults when investigating drinking motives in new settings [32, 33].

Research on drinking motives and motives to limit drinking among HIV-positive adults in Africa is needed to design effective interventions to support individuals in reducing their alcohol intake. Accurate information on how and why persons with HIV drink or abstain, and how alcohol use is being managed in HIV treatment settings, could make a significant contribution to ancillary care for HIV/AIDS in Uganda and elsewhere [34]. To this end, we conducted a qualitative study of changes in alcohol use among HIV-infected Ugandan adults during their first year of HIV care.

## Methods

### Study Design

The qualitative research reported here was carried out as part of a larger, prospective cohort study of changes in alcohol consumption in adults during the first year of care and treatment at a major HIV specialty clinic in rural southwest Uganda. The goal of the qualitative study was to characterize in-depth the circumstances surrounding change in alcohol consumption for a subset of cohort study participants. Four specific questions were addressed in the qualitative work: (1) how consumption of alcohol was addressed by clinic staff in conversations with patients as they entered HIV care; (2) circumstances under which patients used alcohol; (3) how patients worked to change their alcohol consumption over time; and (4) what social contextual and psychological factors they described as influencing their attempts at change.

The research was approved by the Committee on Human Studies, Harvard Medical School, Boston, MA; the Committee on Human Research, University of California at San Francisco, San Francisco, CA; the Institutional Ethical Review Committee, Mbarara University of Science and Technology, Mbarara, Uganda; and the Uganda National Council for Science and Technology, Kampala, Uganda.

### Study Setting

The study took place at the immune suppression syndrome (ISS) Clinic in Mbarara, Uganda, a large town approximately 300 km southwest of the capital city of Kampala. The ISS Clinic is an HIV/AIDS treatment and research facility nested within the regional referral hospital for rural southwest Uganda. The clinic provides antiretroviral treatment and comprehensive care for HIV-infected adults and children. At the time of the study, it was staffed by eight physicians and medical officers, four nurses and five counselors. Ancillary staff consisted of five laboratory technicians, four pharmacists, eleven data and records clerks, four health educators, two paid patient trackers, and a number of volunteers. Nearly 10,000 adults were being followed, 88 % of whom were using ART. On average, 110 adults were initiating ART each month.

### Sampling and Recruitment

Three thousand seven hundred and forty-seven HIV-infected adults were screened for enrollment in the prospective cohort study as they entered into HIV care. Of these, 1,246 (33 %) reported using alcohol in the previous year. Study enrollees were adults aged 18 or older who were new patients at the ISS Clinic, reported any alcohol use in the previous year, and had not received HIV care elsewhere. Of those screened, 621 were eligible to participate. Three-hundred-eighty-one individuals were enrolled and randomized to either the cohort (N = 213) or a comparison (N = 168) group that was minimally assessed to examine the impact of regular assessment on research results. Qualitative study participants were randomly selected from the cohort during study enrollment, and referred to qualitative research assistants for recruitment and follow-up. Seventy-one individuals were referred to the qualitative study. Of these, five declined participation, four were lost before enrollment, and three were found to be ineligible after enrollment. Reasons for ineligibility after enrollment were having previously accessed HIV care prior to study enrollment (N = 2) and receiving a false HIV-positive diagnosis (N = 1). The sample for the qualitative study numbered 59 individuals.

### Data Collection

Qualitative study data were collected through direct field observations in the clinic and through individual in-depth interviews with qualitative study participants. Individuals observed were not participants in the qualitative interview study. Consent for observations was obtained verbally from patients and providers at the beginning of each observation session. Written consent to participate in qualitative interviews was obtained from study participants following cohort study enrollment and baseline data collection.

Thirty-eight field observations were carried out between February and June 2011. Field observations focused on patient-provider interactions in the context of clinical activities associated with HIV care. The goal of the observations was to elicit information on how alcohol was addressed by clinic staff in conversations with patients. In addition to patients, physicians, medical officers, nurses, counselors, pharmacists and health educators were observed. Activities observed included enrollment visits, general counseling, ART initiation counseling, adherence counseling, medication dispensing, general health education and follow-up visits with clinicians. Typically, the observer would follow a patient through all of the activities and procedures comprising the visit, or observe shorter clinical interactions with multiple patients. Observation sessions typically lasted two-to-three hours.

Interviews were conducted between July 2012 and February 2014 and were designed to elicit interviewees’ descriptions of (a) alcohol consumption, (b) attempts at reducing or ceasing alcohol consumption, and (c) reasons for drinking and/or attempting to change their drinking habits. They were carried out at a location of the participant’s choosing, in Runyankole, the local language. Interviews were audio-recorded, with permission, and typically lasted about one hour. A total of 164 interviews were carried out at three qualitative data collection points timed to correspond to the baseline, 6 and 12-month data collection points of the larger cohort study. Fifty-nine interviews (N = 59) were completed at Time 1, 53 interviews at Time 2, and 52 at Time 3. Four deaths occurred in the qualitative sample over the data collection period. Three participants were lost to follow-up, for an overall retention rate of 95 % of living participants.

Field observations and in-depth interviews were carried out by Ugandan research assistants trained in methods of conducting qualitative research. Results of observations were written up as field notes, i.e., detailed narrative accounts of what was observed, in English. Interviews were simultaneously translated and transcribed by the research assistants into English. Study data were subjected to quality review for clarity, attention to detail, grammar and style. The total qualitative study data set consisted of 202 written documents: 38 sets of field notes and 164 interview transcripts.

### Data Analysis

Overall, a content analytic approach to the development of descriptive categories was used to analyze the qualitative data [35]. Field notes were reviewed for content describing how alcohol consumption was discussed by clinic staff in conversations with patients. Relevant content was extracted and organized into categories to represent types of information and advice. Interviews were reviewed to develop two coding schemes. One scheme addressed patterns of change in alcohol use, as reported by interviewees. The other represented influences on the success of attempts to change drinking behavior. Following an inductive approach, coding categories were developed for each of the two schemes. The interview transcripts were then coded using each scheme.

#### Patterns of Change Analysis

One-hundred-fifty-six transcripts corresponding to 52 study participants were included in the coding for patterns of change. To maximize validity in the evaluation of change patterns, seven participants for whom fewer than three interviews were available were excluded. Categories describing patterns of change in alcohol use were derived inductively through the repeated reviews of the interview data. Once these categories had been developed and defined, two authors (RS, MW) reviewed the transcripts and assigned each participant to a patterns of change category that in their judgment, best represented the participant’s changes in alcohol use over the course of the study period. Disagreements were resolved through discussion.

#### Influences on Change Analysis

Category development and coding of the interviews for influences on attempts to change drinking behavior were carried out by the primary author (RS). For the “influences” analysis, complete interview transcripts were reviewed for content addressing barriers and facilitators of change attempts. Category labels (“names”) and definitions were developed to sharpen the meaning and scope of each category. Interview excerpts illustrating each category were retrieved from the coded data.

## Results

### Personal Characteristics of the Qualitative Study Sample

Study participants were ART-naive. Most reported being recently identified as HIV-infected—the median number of days since learning of HIV-positive status and enrolling in the larger cohort study was nine (Table 1). The majority of participants (59 %) were male; less than half (46 %) were married. Median age was 30. Slightly more than half (53 %) had attended primary school only; one-third (34 %) had attended secondary school. Based on quantitative data collected at baseline, more than half (56 %) of the group reported consuming alcohol 2–4 times or more per month during the previous three months; 46 % reported drinking 2–3 times per week or more. Forty-four percent of participants reported consuming alcohol once a month or less. Binge drinking was uncommon, but concerning; at baseline, 12 % of study participants reported consuming six or more drinks at a time at least monthly. Six months into care, almost two-thirds (61 %) of those for whom data were available (N = 57) had been prescribed antiretroviral therapy.

**Table 1:** Demographic characteristics of qualitative study participants (N = 59)

Median number of days since HIV diagnosis (N = 57)	9 (IQR = 4–43)
Male N (%)	35 (59 %)
Married N (%)	27 (46 %)
Median age in years	30 (IQR = 26–39)
Educational level N (%)	
None	5 (8 %)
Some primary school	31 (53 %)
Some secondary school	20 (34 %)
Some post-secondary school	3 (5 %)
Alcohol use in preceding three months N (%)	
<1 time/month	26 (44 %)
2–4 times/month	6 (10 %)
2–3 times/week	18 (31 %)
>4 times/week	9 (15 %)
Binge-drinking (six or more drinks at a time) N (%)	7 (12 %)
Prescribed ART at 6 months N (%) (N = 57)	35 (61 %)

### Overview of Qualitative Results

Results of the qualitative analysis are presented below in three sections: (1) how alcohol use was addressed by clinic staff; (2) patterns of change in alcohol use; and (3) influences on change efforts.

1. How alcohol was addressed by clinic staff

Entering into care for HIV/AIDS at the ISS Clinic was a process for patients, involving multiple types of interactions with clinic staff in sequence. The process began with initial HIV testing or confirmatory testing at the clinic lab, after which patients met with a counselor, individually or in a group. In these sessions, counselors provided information about nutrition, prevention of opportunistic infections, safe sexual practices (i.e., condom use), disclosure of HIV status, and alcohol and drug use. As part of the counseling session, patients were instructed to bring a treatment supporter to the next clinic visit. Immediately after counseling, patients met with a nurse, who created their file and recorded intake information. Patients were then sent to a waiting area, where vital signs were taken. The final step was consultation with a clinician (a doctor or medical officer), who may have ordered additional laboratory tests to determine the presence of other infections and confirm ART eligibility. After completing laboratory testing, a follow-up appointment was scheduled.

Observational data indicate that discussions of alcohol use in the ISS Clinic took place largely, but not exclusively, in counseling sessions. Staff emphasized the importance of complete abstinence from alcohol, along with eating well and stopping smoking, as ingredients of a healthy lifestyle. Patients were advised that alcohol is deleterious to health in general and incompatible with antiretroviral medications in particular. Staff warned that alcohol could be detrimental to adherence. Alcohol was also described as undermining the potency of the medications, thus interfering with their beneficial effect. The following excerpts from field notes illustrate:

“There are four things that don’t work alongside these ARVs once you start on them. The first one is alcohol. This alcohol destroys some vital parts of your body that play an important role in the proper functioning of these pills. The alcohol damages them and so it makes it impossible for you to use these pills without much problem…” (Male counselor to male patient, ART initiation counseling session, June 10, 2011)

“Alcohol is not good for you especially when on ARVs. It weakens your body and interferes with the ARVs to work effectively in reducing your viral load.” (Female clinician to male patient, clinician visit, March 31, 2011)

Staff did not offer explanations of the biological mechanisms through which alcohol was considered to negatively impact ART, nor did they recommend anything short of abstinence. Suggestions that reducing amounts of alcohol intake might also be beneficial, for example, did not figure into counseling advice.

Strategies for motivating patients to stop using alcohol included encouraging them to consider the impact of drinking on their hopes and plans for the future and/or to focus on fulfilling financial responsibilities to family, as we see in the following:

“Alcohol is not a solution to any problems. It actually makes them worse. You only need to re-think about the life aspirations you had and this may give you hope for future.” (Female counselor to male patient, adherence counseling session, May 30, 2011)

“I will be happy for you to pick yourself together and start working hard to sustain your family rather than looking at yourself with pity. All this will be possible if you make your mind up to take a job, no matter how small, leave this alcohol behind and take your pills properly. I will await your success story.” (Male counselor to male patient, adherence counseling session, June 17, 2011)

2. Patterns of change in alcohol use

Participants responded to alcohol-related advice and admonitions received from counselors in a variety of ways. We identified five patterns of change in alcohol use over the qualitative study period. They are: *cessation,* *reduction*, *no change*, *increase* and *fluctuation*. Cessation involves eventual complete abstinence from alcohol. Reduction is characterized by reported decreases in amount and/or frequency of alcohol intake following the initial baseline interview, stopping short of complete abstinence. No change in alcohol use refers to a stable pattern of consumption over the data collection period of the qualitative study. Increase means that self-reported amount of alcohol intake and/or drinking frequency increased over the course of the study. Fluctuation describes an inconsistent pattern of reported frequency of alcohol use and/or quantity of alcohol consumed over the course of the interviews.

Results suggest the majority of participants attempted to cease or reduce alcohol consumption following entry into HIV care. Fifteen participants (29 %) fit the “cessation” pattern, reporting having stopped using alcohol completely (Table 2). Eighteen participants (35 %) were classified as having reduced, but not ceased, alcohol use. Almost half of the participants who met the criteria for reducing or ceasing alcohol use were rare or infrequent drinkers (less than once per month at baseline, as reported on the quantitative interview); the other half consumed alcohol regularly (two to four times per month) or frequently (2 times per week or more) prior to baseline.

**Table 2:** Patterns of change in alcohol use over one year, by frequency of drinking at baseline (N = 52)

Pattern of change category	Overall	Rare/infrequent drinkers	Regular/frequent drinkers
Cessation	15 (29 %)	7 (30 %)	8 (28 %)
Reduction	18 (35 %)	8 (35 %)	10 (35 %)
Fluctuation	9 (17 %)	2 (9 %)	7 (24 %)
Increase	1 (2 %)	0 (0 %)	1 (3 %)
No change	9 (17 %)	6 (26 %)	3 (10 %)
Total	52	23	29

Seventeen percent of the sample (N = 9) were characterized as showing “no change” in the frequency of alcohol use or quantities consumed. Participants in this category included both frequent drinkers who consumed alcohol at least twice per week, as well as individuals who reported using alcohol rarely or infrequently. Rare drinkers in this category reported stopping alcohol use before initiating HIV care, and did not resume alcohol consumption during the study. Only one individual appeared to steadily increase alcohol use over the course of the study.

Seventeen percent (N = 9) of participants evidenced fluctuating patterns of drinking. Fluctuation described changing patterns of use, including multiple cycles of increasing and/or decreasing alcohol consumption or frequency over the course of the study. Periods of stable consumption or abstinence were sometimes interspersed among otherwise fluctuating patterns of use. Fluctuation was mostly represented in individuals who drank regularly or frequently but was also reported in rare and infrequent drinkers (See Table 2).

3. Influences on change efforts

Results reporting influences on change efforts described by study participants are organized into three general categories reflecting: (I) reasons to reduce or abstain from using alcohol; (II) incentives to drink alcohol; (III) moral conflicts. Reasons to reduce or abstain included: (a) advice against drinking from clinicians; (b) interference with fulfillment of social obligations; (c) threats to financial security; and (d) threats to social standing in the community. Incentives to drink alcohol included: (e) social benefits of drinking, including protection against disclosure of one’s HIV status; (f) alcohol as stress relief; and (g) pleasure and enjoyment of using alcohol. Individuals experienced reasons to reduce drinking and incentives as competing influences, generating moral conflicts and making them feel caught in a “tug of war.” No gender differences in patterns of change or influences on change were apparent.

- Reasons to reduce or abstain from alcohol use
- Advice against alcohol use from clinicians

As part of initiating HIV care at the ISS Clinic, staff advised patients to abstain from using alcohol on the grounds that drinking would interfere with antiretroviral therapy, and in general be detrimental to health. Such admonitions resonated strongly with participants, some of whom, feeling ill, had ceased alcohol use prior to enrolling in care. Knowing that ART could be life-saving, and fearing accelerated death through non-adherence or medication ineffectiveness, participants took such clinical advice to heart. One participant explained that she did not want to risk reducing the effectiveness of her medications, and therefore had abstained from alcohol following her enrollment in care:

“I have not taken alcohol because I started taking ARVs. The doctors advised me not take alcohol or cigarettes anymore to facilitate proper functioning of these pills. The doctors also told me that if the drug is to work 100 %, it can end up reducing its performance to like 10 % if one continues to drink alcohol. I would be unwise if I continued taking it even when a doctor has insisted it is bad. I would feel unhappy wasting my time taking these hard pills only to be told they did not function properly after all because of the stupid alcohol.” (Female, Age 21, Interview 2)

b. Interference with fulfillment of social obligations

While counselors emphasized the adverse physical effects of continued alcohol use, participants themselves pointed to the negative impact of alcohol on their ability to fulfill social obligations, particularly towards their children. Children were left unattended when a parent was away from home consuming alcohol. Children suffered when parents spent household funds on alcohol rather than school fees. A participant described his sense of shame when he recalled how his children actually missed school as a result of his having run short of money due to drinking:

“I once drank alcohol and failed to pay my children’s fees. They were sent home for about two weeks and almost missed their exams. That has never left my mind … I had bought a lot of alcohol and drank it with my friends. That was the money I had saved for my daughter’s school fees and I never got to forgive myself for that.” (Male, Age 37, Interview 3)

Alcohol use was also implicated as a cause of strain on relationships between spouses, and with other family members and friends. Alcohol precipitated arguments and infidelity. Alcohol intoxication was also reported to result in episodes of physical or verbal abuse, where spouses, family and friends could fall victim. In the following example, a participant recalled regretfully how alcohol contributed to an altercation with her sister:

“I went out to drink with my sister and became drunk. I got drunk and abused my sister who sheltered me for a long time. I abused her seriously and I felt very guilty the next morning when she confronted me about it.” (Female, Age 21, Interview 2)

c. Threats to financial security

In resource-scarce environments, where people are poor, spending money preferentially on one commodity may mean doing without another. Thus, money spent on alcohol may interfere with participants’ abilities to meet basic household obligations, such as obtaining food or paying rent. Sometimes, possessions are sold to finance alcohol use. Over the long term, prioritizing alcohol can become a roadblock to social mobility and improved quality of life, as households lack the capital to invest in land or pursue educational opportunities. The relationship between alcohol use and impoverishment is illustrated in the following case:

“I am very poor today because of alcohol. Maybe I would have been able to save money and buy myself land or build a house. I even had to sell off the small land I inherited from my father to take more alcohol. I have wasted away my life … I think if I manage to stop this alcohol thing, I should be able to save some money and buy some land.” (Male, Age 41, Interview 1)

d. Threats to social standing

Social capital is a resource grounded in and stemming from networks of relationships [36]. Being in good standing in these networks is central to achieving desired ends. In African social settings, social capital is often brought to bear in overcoming economic obstacles to ART adherence and persisting in HIV care [37]. Alcohol intoxication may threaten social capital by resulting in behavior considered incompatible with cultural definitions of personal responsibility. Episodes of drunkenness led to interpersonal conflicts or “embarrassing” behavior that can damage social relationships, lowering social standing and jeopardizing social capital. Social standing is also compromised when alcohol use leads to negative judgments by others. The following excerpt illustrates concern for social status in the context of alcohol use:

“It is embarrassing. I am a woman and it is embarrassing if you are to be found drunk in a bar all the time, like every day. It is not good … People can think you are irresponsible. I mean, you leave your family and children at home only to come back drunk? It is so irresponsible and embarrassing, especially in this village. You will definitely get people talking.” (Female, Age 20, Interview 1)

II. Incentives to drink alcohol

Despite reasons to the contrary, HIV-infected patients initiating HIV care and treatment at the ISS Clinic described numerous incentives to continue alcohol use.

e. Social benefits of drinking

Alcohol use reinforces social relationships and facilitates inclusion within a peer group. Both men and women described consumption of alcohol as a central part of gender-specific social activities and peer-group bonding. Drinking alcohol in a peer group also fulfilled social expectations of reciprocity and politeness. Turning down an offer of alcohol was considered rude, as peers “insisted” on purchasing drinks, and expected reciprocity. Interviewees found these offers difficult to decline for fear of losing social standing. One participant described his struggle to abstain from alcohol and face the possibility of ostracism from his peers:

“It’s not easy because a thief walks in a company of a thief. A drunkard walks and enjoys a company of a drunkard, so if you don’t do what they do, they distance themselves from you slowly. This is because whenever they call you and buy for you a few beers, they expect you to buy for them the next round.” (Male, Age 30, Interview 2)

Refusal of alcohol is considered a marker of illness, and some participants were reluctant to pursue abstinence out of fear others might then suspect they were HIV-infected. The apprehension that unexplained abstinence would be interpreted as evidence of being HIV-infected is reflected in the following citation, in which an interviewee is searching for a socially acceptable way of explaining to her friends why she is not drinking:

“[My friends] could keep pressuring me to take some alcohol and I am sort of looking for a way to handle this. It’s like, you can’t refuse just like that. I mean you ought to have a reason for your decision and maybe joke about it, or otherwise they could start suspecting something.” (Female, Age 21, Interview 1)

f. Alcohol as stress relief

Some interviewees characterized alcohol as important in dealing with social stressors and helping to facilitate a restful night’s sleep. Drinking alcohol was described as a means of “dulling” or “forgetting” one’s problems, in effect, turning off the mind as a way of gaining peace. One interviewee described the perceived calming benefits of alcohol consumption this way:

“If you have got many thoughts, or an issue disturbing you, [alcohol] helps you to forget the problems and helps your brain to focus on something else.” (Female, Age 21, Interview 1)

Participants also found it challenging to abstain from alcohol in the context of a new HIV diagnosis, as this itself may bring significant emotional turmoil. One individual described the role of alcohol in his life as he struggled to come to terms with being HIV-infected:

“I was tested and found HIV positive, so what more is there? … I have not yet married and I do not want my family members to know about my status. Why would I want to produce a child to leave for my mother when I die? So, I wanted to forget the things of this world [by drinking]… When I get sober, I start thinking about those things again.” (Male, Age 30, Interview 1)

g. Pleasure and enjoyment of using alcohol

Alcohol was often considered a central component of social gatherings during holidays and celebrations. Interviewees also reported that they “yearn for” and enjoy the taste of alcohol itself. Drinking alcohol with peers “for fun” was described as the “good life.” Enjoyment of drinking made abstinence more difficult. One patient participant described the pleasure associated with alcohol use:

“Of course, you feel good when you are drinking [alcohol] and eating roasted meat. What else would you want? By the way, that’s heaven on earth.” (Male, Age 40, Interview 1)

III. Moral conflicts: The “tug of war”

Arguments against use of alcohol weighed against incentives to continue drinking for this group of HIV-infected individuals. As a result, some felt caught in a “tug of war” pitting the ‘push’ of preserved health and social responsibility as desired ends against the ‘pull’ of maintaining social connectedness and experiencing the pleasures of alcohol. While participants may have been instilled with good intentions to abstain as a result of their counseling experiences, they found it difficult to translate this into practice. The resulting “tug of war” posed a moral quandary for these participants, as individuals struggled to make “good” decisions in a sea of “bad” influences. The following examples demonstrate how participants felt caught between these opposing forces:

“Sometimes it is just because of peer pressure. I sometimes feel like going to the trading center and when I get there, the other girlfriends suggest that we go for a drink, and that is it. You know, bad company corrupts good character.” (Female, Age 41, Interview 1)

“I even told my sister that I was not going to take any alcohol because I had already been started on Septrin … She insisted and asked me why I had accepted to come along with her. I felt out of place and so sent for a beer though I knew I was doing something bad.” (Female, Age 41, Interview 1)

This moral struggle is dynamic and ongoing. Though these participants may have been able to decrease or cease alcohol use in the short term, they recognized that factors encouraging use were never going to be completely absent from their lives. One participant laments about this ongoing conflict in his household and the potential challenges he will face in the future:

“It is very bad if you are trying to stop drinking and there is this other person in your house who wants to drink every day. It is challenging … I promised the doctor that I will not be drinking again but we all have challenges in life … You avoid alcohol but you can’t avoid it forever, especially when you are to find it at home waiting there.” (Male, Age 27, Interview 2)

## Discussion

The goal of this qualitative study was to examine how HIV-infected persons entering into care and treatment for HIV worked to change their alcohol use over time. We examined patterns of change as described in qualitative interviews, as well the impact of social and psychological factors on efforts to reduce alcohol intake. A majority of study participants appeared to cease or reduce alcohol intake over the study period. Some also returned to or increased their drinking again. Reasons for decreasing or ceasing alcohol consumption included: (a) advice against drinking received from clinic staff; (b) interference with fulfillment of social obligations; (c) negative impact on financial security; and (d) threatened social standing in the community. Competing with these reasons were a number of incentives to drink, including reinforcement of social relationships, stress relief, and overall pleasure and enjoyment gained from alcohol consumption. The result was a “moral tug of war”—with some participants trying to balance the “pull” of perceived benefits of drinking with the “push” of negative consequences from continued alcohol use.

Notably, we identified three influences on patterns of alcohol use related to HIV-infected status. Participants responded strongly to hearing from clinicians that continued alcohol use would undermine the effectiveness of antiretroviral therapy. Conversely, some found abstinence challenging while coping with the emotional turmoil brought on by their new HIV diagnosis. In addition, a fear that sudden unexplained abstinence might result in attributions of HIV-infected status by others contributed to the “pull” toward continued drinking. Otherwise, influences on efforts at changing drinking behavior appeared not to be directly related to one’s HIV status.

In fact, most of the influences on drinking behavior identified for this HIV-infected, rural Ugandan population appear similar to those described for general populations in resource-rich locations. Using alcohol to facilitate social inclusion, cope with stress, and enhance pleasure have been reported repeatedly in studies carried out across age ranges in the US [28–31]. Documentation of cross-cultural similarities in the reasons people drink and/or try to cut down or stop has implications for thinking about how to intervene on hazardous alcohol use in the Ugandan setting, i.e., interventions that have been effective in other populations may be useful in reducing alcohol consumption in persons with HIV in Uganda as well.

The “tug of war” some participants experienced between competing influences on drinking suggests ambivalence. Ambivalence is a common experience among heavy alcohol consumers, particularly those who are seen in medical settings [38]. The “conflict between indulgence and restraint” is explored and leveraged as a key step in behavior change in the Motivational Interviewing (MI) approach to reducing alcohol consumption. MI has received extensive research attention in the US [39]. A brief version of MI (BMI) has shown efficacy for heavy drinkers in a variety of settings [40].

BMI is a key component of a number of screening and brief interventions (SBI). Brief interventions (BI) for alcohol use have been shown to be efficacious in reducing unhealthy alcohol use in primary care settings in the US and Europe [41]. Results of experimental studies of SBI carried out in South Africa have been less definitive [42–44]. Brief interventions typically include feedback on drinking levels, emphasis on personal responsibility, advice on drinking, consideration of options for change, and promotion of self-efficacy. They range in length from 1 to 50 min and are delivered in one or two sessions [41] and thus may be feasible in resource limited settings. Results of this qualitative study suggest that BI that include BMI may be feasible and effective interventions to reduce alcohol use among individuals living with HIV in Uganda.

This study has limitations that should be noted here. Our characterizations of patterns of change in alcohol use are based on participants’ narrative accounts of their drinking behaviors provided in qualitative interviews. The likely influence of social desirability on participants’ representations of their drinking must be acknowledged. Alcohol use is a highly stigmatized behavior in this setting and participants were being cautioned explicitly against using alcohol by clinic staff as part of providing them with life-saving antiretroviral medication for HIV/AIDS. In collecting data from participants, qualitative study staff worked to clearly distinguish themselves from clinic staff providing them with care. Nonetheless, it was clear in some cases that interviewees perceived us as “wanting them to stop drinking.” These perceptions may have shaped how they described their alcohol consumption; thus, in some cases under-reporting is likely. However, the overall trends of decreasing alcohol consumption around the time of entry into HIV care found in this study are consistent with results from the larger quantitative cohort study, in which alcohol consumption was measured using a combined measure of hazardous drinking (self-reported hazardous drinking and phosphatidylethanol, a biomarker of alcohol consumption) [45, 46].

## Conclusion

HIV-infected adults in rural southwest Uganda entering into care and treatment for HIV struggle with conflicting pressures in responding to clinical advice to abstain from alcohol use. Without additional support, they may not succeed in making long-term changes in their drinking habits. A brief intervention that addresses the obstacles to change patients themselves have identified and that can be implemented by existing clinic staff with periodic “boosters” to reinforce patients’ efforts could be helpful in bringing long-term reductions in alcohol use. Identifying and adapting such an intervention, and investigating its efficacy, is a natural next step in improving care and support for HIV-infected Ugandans working to preserve their overall health. Future research should include determination of ideal steps for implementation of effective alcohol interventions that will succeed in both initiating and sustaining behavior change in resource limited settings.
